# Infection of *Mycobacterium tuberculosis* Promotes Both M1/M2 Polarization and MMP Production in Cigarette Smoke-Exposed Macrophages

**DOI:** 10.3389/fimmu.2020.01902

**Published:** 2020-08-20

**Authors:** Yanqing Le, Wenli Cao, Lu Zhou, Xin Fan, Qiangui Liu, Fusheng Liu, Xiaoyan Gai, Chun Chang, Jing Xiong, Yafei Rao, Aling Li, Wei Xu, Beibei Liu, Tong Wang, Beinan Wang, Yongchang Sun

**Affiliations:** ^1^Department of Respiratory and Critical Care Medicine, Peking University Third Hospital, Beijing, China; ^2^Beijing Geriatric Hospital, Beijing, China; ^3^CAS Key Laboratory of Pathogenic Microbiology and Immunology, Institute of Microbiology, Chinese Academy of Sciences, Beijing, China; ^4^Department of Thoracic Surgery, Peking University Third Hospital, Beijing, China

**Keywords:** chronic obstructive pulmonary disease, cigarette smoke, *Mycobacterium tuberculosis*, macrophage polarization, matrix metalloproteinase

## Abstract

Pulmonary tuberculosis (PTB) is a risk factor for COPD. Our previous study revealed more severe emphysema in COPD patients (mostly smokers) with prior tuberculosis. However, the mechanisms of interactions between cigarette smoke (CS) and *Mycobacterium tuberculosis* (Mtb) are unknown. In this study, we found that the frequencies of both M1 and M2 macrophages, and levels of MMP9 and MMP12 in bronchoalveolar lavage were increased in PTB patients with smoking. Between-group analysis showed that the frequency of M1 macrophages was higher in non-smoker PTB patients while more M2 macrophages were found in smokers without PTB, as compared to the non-smoker healthy controls. Bacille Calmette-Guérin (BCG) infection in CS extract (CSE)-incubated MH-S cells further enhanced secretion of M1-related (iNOS, IFN-γ and TNF-α) and M2-related (TGF-β and IL-10) cytokines, reactive oxygen species (ROS) production and cellular apoptosis, concomitantly with up-regulation of MMP9 and MMP12, but not TIMP1. Moreover, BCG infection in acutely CS-exposed mice promoted macrophage polarization toward both M1 and M2 phenotypes, along with increased lung inflammatory infiltration. MMP9 and MMP12, but not TIMP1, were further up-regulated in lung tissues and BAL fluid after BCG infection in this model. Taken together, Mtb Infection promoted CS-exposed macrophages to polarize toward both M1 and M2 phenotypes, along with enhanced production of MMP9 and MMP12. These findings provide insights into the mechanistic interplay between CS exposure and tuberculosis in the pathogenesis of COPD.

## Introduction

Chronic obstructive pulmonary disease (COPD) is a chronic airway disease characterized by persistent inflammation and lung tissue destruction. Cigarette smoking (CS) is the major risk factor for COPD (1), but studies have also found that tuberculosis is associated with increased risk of COPD, especially in regions with a high disease burden (2–5). Our previous study showed that COPD (mostly smokers) with prior tuberculosis had severe emphysema and a higher prevalence of bronchiectasis (6). However, the mechanisms of interactions between cigarette smoke (CS) and *M. tuberculosis* (Mtb) in the development of COPD are unknown.

Macrophages, as innate immune cells, play a critical role in defending against exogenous pathogens and resolution of inflammation, contributing to lung homeostasis (7). Studies in humans and animal models have underlined the critical role of macrophages in the inflammatory process and lung tissue destruction in COPD (8, 9). Macrophages are classified into classically activated (M1) and alternatively activated (M2) phenotypes. M1 macrophages are induced by interferon γ (IFN-γ) and lipopolysaccharide (LPS), and produce inducible nitric oxide synthase (iNOS), reactive oxygen species (ROS) and cytokines such as IFN-γ, interleukin 1β (IL-1β) and tumor necrosis factor α (TNF-α), exerting pro-inflammatory and cytotoxic effects. M2 macrophages are stimulated by interleukin 4 (IL-4), interleukin 10 (IL-10), and interleukin 13 (IL-13), and secrete IL-10 and transforming growth factor-β (TGF-β), involving in immune-regulatory and tissue-remodeling processes (7, 10, 11). Studies have indicated that macrophage polarization is closely related to the pathogenesis of COPD or pulmonary tuberculosis (PTB) (12–14). M1- polarized and M2- polarized lung macrophages increased significantly with smoking and COPD severity (12). While in Mtb infection, the M1 phenotype was upregulated in the early phase of disease, but in the intermediate and late phases the M2 phenotype dominated (13). Therefore, a better understanding of macrophage polarization is essential to unveil the interplay between CS exposure and Mtb in leading to excess lung destruction in COPD.

Matrix metalloproteinase (MMP), proteolytic enzymes capable of degrading extracellular matrix (ECM) such as elastin and collagen (15), are intricately involved in the inflammatory response and tissue restructuring that play critical roles in the pathogenesis of different lung diseases (16, 17). A growing body of evidence indicates that MMP9 and MMP12, largely secreted from macrophages, are implicated in the breakdown of lung tissues in COPD and emphysema (18–20). An interesting study by Ishii et al. indicated differential roles of MMP12 and MMP9 in COPD, MMP12 in the pathogenesis of emphysema, while MMP9 in the development of airflow limitation (21). Besides, increased MMP production and activity, including MMP9 and MMP12, were found to be involved in lung inflammation and tissue destruction caused by PTB (22, 23).

Thus, we hypothesized that CS exposure and Mtb infection would lead to abnormal M1/M2 polarization and MMP production in macrophages, which were therefore involved in enhanced inflammation and tissue destruction seen in COPD associated with PTB.

## Materials and Methods

### Study Subjects

Forty-seven subjects, including PTB patients with current cigarette smoking (PTB+CS group), PTB patients without smoking history (PTB group), Smokers (CS group) and Non-smokers without PTB (Normal control), were consecutively enrolled in Peking University Third Hospital and Beijing Geriatric Hospital. Written informed consent was obtained from all human subjects and the Ethics Committee of Peking University Third Hospital and Beijing Geriatric Hospital approved this study. Smokers were subjects with a smoking history of ≥10 pack-years and post-bronchodilator FEV1/FVC ≥ 70%. Patients aged 18 years and older are eligible for inclusion if they are diagnosed with PTB by the tuberculosis diagnostic criteria in Health Industry Standards of the People's Republic of China. The PTB patients had a negative sputum smear or culture or converted from positive to negative after receiving anti-tuberculosis therapy for ≥3 months. Patients in the Normal Control group denied smoking history, had a normal lung function (post-bronchodilator FEV1/FVC ≥ 70%), and received bronchoscopy for a solitary lung nodule. Patients with malignant tumors, interstitial lung diseases, autoimmune diseases or other immune-related diseases were excluded.

### Flow Cytometric (FCM) Analysis

Bronchoalveolar lavage (BAL) samples were obtained by using a standardized protocol of fiberoptic bronchoscopy and filtered through nylon gauze (24). After centrifuged for 10 min at 400 g at 4°C, cells were resuspended in fluorescence-activated cell sorter (FACS) buffer (PBS supplemented with 0.5% bovine serum albumin and 2 mM EDTA). Cells were stained with conjugated monoclonal antibodies to detect membrane markers. To further examine intracellular markers, cells were subsequently fixed with 4% paraformaldehyde (PFA) fixation for 40 min and permeabilized with Fixation and Permeabilization Solution (BD) for 20 min, and stained with conjugated antibodies to detect intracellular markers. The following human antibodies were applied for BAL samples: anti-CD68 FITC (eBiosciences), anti-CD86 PE (eBiosciences), anti-CD163 APC (eBiosciences). The cells from BAL were not stained with anti-CD16/CD32 antibody for blocking Fc receptors in this analysis. Besides, MH-S cells were treated with stimulus, and were staining the following mouse antibodies: anti-p-Stat5 FITC (eBiosciences) and anti-p-Stat3 PE (eBiosciences). The staining cells were measured through Canto Flowcytometer (BD Biosciences). Data were analyzed using FlowJo 7.6 (BD) software. Isotype controls and fluorescence minus one control were used to set gates.

### Measurement of MMP9, MMP12, and TIMP1 in BAL Fluid and Serum

BAL fluid and serum were collected and used for assessing MMP9, MMP12, and TIMP1 levels by enzyme-linked immunosorbent assay (ELISA), according to the manufacturer's instructions for ELISA kits (Elabscience, Wuhan, China).

### CS Extracts (CSE) Preparation

The preparation of CSE was performed as described in our previous study (25). Briefly, smoke fog from five cigarettes (Bai Sha) was bubbled through 10 ml of RPMI-1640 medium at a constant airflow and then was sterile filtered through a 0.22 μm filter (Millipore). Absorbance value was constant (4.0 ± 0.05) by measuring at a wavelength of 320 nm.

### Cell Culture and Treatment

The mouse alveolar macrophage cell lines (MH-S cells) were purchased from Bio-Rad Biological Technology Co. Ltd. (Shanghai, China), an agent of ATCC cells. Cells were incubated in the incubator under 37°C and 5% CO_2_ conditions. RPMI-1640 medium (Hyclone) containing 10% fetal calf serum (Gibco) was changed every day and then MH-S cells were seeded to proper culture plates at a density of 10^5^cells/ml. CSE and/or BCG were then added to the culture medium for the following experiments.

### Cell Viability by MTT Assay

Cell viability was tested with the MTT assay according to the manufacturer's instructions. In brief, cells were seeded into 96-well plates at a density of 2,000 cells/well. Absorbance was read in a spectrophotometer at a wavelength of 570 nm.

### Western Blot for MMP9, MMP12, and TIMP1 Expressions

To assess the protein expressions of MMP9, MMP12, and TIMP1 in incubated cells, cellular protein extracts were added to 10% SDS-PAGE, transferred to 0.22 μm PVDF membrane (Millipore) and the membranes were incubated overnight with antibodies as follows: MMP9 (1:500), MMP12 (1:500), and TIMP1 (1:500). Then membranes were incubated with HRP-conjugated donkey anti-rabbit or anti-mouse IgG antibody (1:1,000, CST) for 1 h at room temperature and then visualized by enhanced chemiluminescence (Millipore). Quantitative image analysis was performed by using Image J software.

### Measurement of mRNA Expression by Real-Time Quantitative PCR

Real-time quantitative PCR (qPCR) method was used for the determination of the differential gene expressions of MMP9, MMP12, TIMP1, iNOS, IFN-γ, TNF-α, TGF-β, and IL-10 in MH-S cells. Total cellular RNA was isolated from MH-S cells using TRIzol reagent (Thermo Fisher Scientic), and cDNA was synthesized using the PrimeScript RT Reagent Kit (Takara). The qPCR reactions were performed on the Bio-Rad CFX Manager in a 20 μL reaction system by using SYBR Green One-Step qRT-PCR Kit (Tiangen, Beijing, China). The primer sequences were as follows: GADPH: 5′-AAATGGTGAAGGTCGGTGTGAAC-3′(sense) and 5′-CAACAATCTCCACTTTGCCACTG-3′(antisense); MMP9: 5′-CGTGTCTGGAGATTCGACTTGA-3′(sense) and 5′-TGGTTCACCTCATGGTCCAC-3′(antisense); MMP12: 5′-TGGTACACTAGCCCATGCTTT-3′(sense) and 5′-AGTCCACGTTTCTGCCTCATC-3′(an-tisense); TIMP1: 5′-TACACCCCAGTCATGGAAAGC-3′(sense) and 5′-CGGCCCGTGATGAGAAACT-3′(antisense); iNOS: 5′-ACCAGAGGACCCAGAGACAA-3′(sense) and 5′-CCTGGCCAGATGTTCCTCTA-3′(antisense); IFN-γ: 5′-TGAACGCTACACACTGCATCTTGG-3′(sense) and 5′-TGACTCCTTTTCCGCTTCCTGAG-3′(antisense); TNF-α: 5′-GGCAGGTCTACTTTGGAGTCATTGC-3′(sense) and 5′-ACATTCGAGGCTCCAGTGAATTCGG-3′(antisense); TGF-β: 5′-ACGTCACTGGAGTTGTACGG-3′(sense) and 5′-GGGGCTGATCCCGTTGATT-3′(antisense); IL-10: 5′-ACCTGGTAGAAGTGATGCCCCAGGC A-3′(sense) and 5′-CTATGCAGTTGATGAAGATGTCAAA-3′(antisense). The cycling conditions used were 95°C for 10 s, 60°C for 32 s, for 40 cycles, followed by a melting point determination or dissociation curves. Relative gene expression to control was calculated using the 2^−ΔΔCT^ method.

### ROS Measurement

The intercellular ROS level was measured by 2, 7-Dichlorodihydrofluorescein diacetate (DCFH-DA) method. In brief, after MH-S cells were treated with CSE and/ or BCG, cells were further incubated with 10 mM DCFH-DA (Sigma-Aldrich) for 30 min at 37°C. After washed three times with ice-cold phosphate buffer saline (PBS), cells were harvested and kept on ice for immediate detection by FCM.

### Apoptosis Measurement

Cell apoptosis was measured by Dead Cell Apoptosis Kit with Annexin V FITC and PI (Thermo Fisher Scientic), according to the manufacturer's instructions. In brief, MH-S cells were treated with CSE and/or BCG, and then collected and co-cultured with propidium iodide (PI) and FITC-labeled Annexin V for 15 min at 37°C. After washed three times with ice-cold PBS, cells were kept on ice for immediate detection by FCM.

### Cigarette Smoke Exposure Protocol for the Mouse Model

Procedures for establishment of the mouse model were approved by the Ethics Committee of Peking University Third Hospital and followed the committee's animal care and use guidelines. Intranasal methods of CSE exposure or BCG infection were used (26, 27). Briefly, 6–8 weeks old, female, C57BL/6 mice (specific-pathogen-free) were purchased from Beijing Vital River Laboratory. Mice were exposed to CSE (40 μl) or PBS by intranasal method for 7 consecutive days, and then infected with Bacille Calmette-Guérin (BCG) (0.5 × 10^7^ in 40 μl volume) by intranasal method instillation at 7th day and sacrificed after 3 days. After inserting a catheter (24 G needle) into the trachea, BAL samples were collected after instillation of PBS solution (0.6 ml × 2) through a needle. Lung tissues from mice without lavage were removed, and the left lung was inflated with formalin and paraffinized for histological staining while the right lung was frozen by liquid nitrogen for protein measurements.

### Immunohistochemistry and Immunofluorescence of Lung Tissues

Lung tissue sections (5 μm thick) were deparaffinized, and treated with 3% H_2_O_2_-CH_3_OH for 15 min to block endogenous peroxidase. Samples were submerged in citrate buffer (pH 6.0) in a microwave oven for antigen retrieval, blocked with 5% BSA for 30 min at room temperature and then incubated overnight with antibody F4/80 (1:1000), iNOS (1:100), CD163 (1:50), MMP9 (1:100), and MMP12 (1:100). After washing with PBS, slices were incubated with horseradish peroxidase (HRP)-conjugated goat anti-rabbit IgG (ZSGB-Bio, Beijing, China) at 37°C for 30 min and then stained with DAB detection system kit (ZSGB-Bio). The expression and localization of these molecules in the lung were detected under light microscopy and analyzed by image-pro plus 6.0 software.

For immunofluorescence, lung tissue sections were co-incubated overnight with antibodies F4/80 (1:1,000) with iNOS (1:100) or CD163 (1:100). After washing with PBS, sections were incubated with alexa 594-labeled goat anti-rabbit secondary antibody and alexa 488-labeled goat anti-mouse/rat secondary antibody for 1 h at 37 °C and then 4, 6-diamidino-2-phenylindole dihydrochloride (DAPI) (Beyotime, Shanghai, China) were added for cellular nuclear staining. Co-localization of F4/80 and iNOS or CD163 was evaluated with confocal microscopy (TCS-SP8; Leica Microsystems, Wetzlar, Germany).

### Statistical Analysis

All the data were expressed as mean ± standard deviation (SD) and analyzed using SPSS 20.0 software. Two-group comparisons were performed using Student's *t*-test. Three or more group comparisons were performed with one-way analysis of variance (ANOVA) accompanied by Bonferroni *post hoc* test (equal variances assumed) or Dunnett's T3 (equal variances not assumed) *post hoc* tests. Values of *P* < 0.05 were considered to be statistically significant.

## Results

### Demographic Characteristics of Study Population

The study included 10 smokers with PTB (PTB+CS group), 16 non-smokers with PTB (PTB group), 10 smokers without PTB (CS group), and 11 non-smoking subjects without PTB (Normal control). Table 1 shows the clinical characteristics of these subjects. The differences in age and post-bronchodilator FEV1/FVC were significant among groups.

**Table 1:** Demographic characteristics of study population.

	**Normal control**	**CS group**	**PTB group**	**PTB+CS group**	***p*-value**
Subjects (*n*)	11	10	16	10	
Age (Years)	59.58 ± 13.92	57.70 ± 7.47	29.12 ± 8.49	47.56 ± 13.02	<0.05
Gender (male/female)	5/6	10/0	11/5	9/1	
BMI (kg/m^2^)	23.56 ± 3.04	24.47 ± 3.54	24.47 ± 3.57	22.82 ± 2.48	NS
Smoking index	0	26.90 ± 7.90	0	21.33 ± 5.89	
(pack-years)					
Anti-tuberculosis (Months)	0	0	8.42 ± 4.21	9.11 ± 3.82	
FEV1 %pred	87.15 ± 6.90	89.96 ± 9.75	94.55 ± 11.89	91.33 ± 12.06	NS
Post-bronchodilator FEV1/FVC (%)	84.06 ± 7.40	76.78 ± 4.95	88.82 ± 4.03	73.95 ± 5.75	<0.05

*Smoking index, average number of cigarettes per day (pack) × number of years of smoking history (years); BMI, weight (kilograms, kg)/square of height (square of meters, m^2^). Mean ± SD*.

*BMI, body mass index; FEV1, forced expiratory volume in 1 s; FVC, forced vital capacity*.

### Increased M1 and M2 Polarization and MMP Production in PTB Patients With Smoking

**Figure 1:**
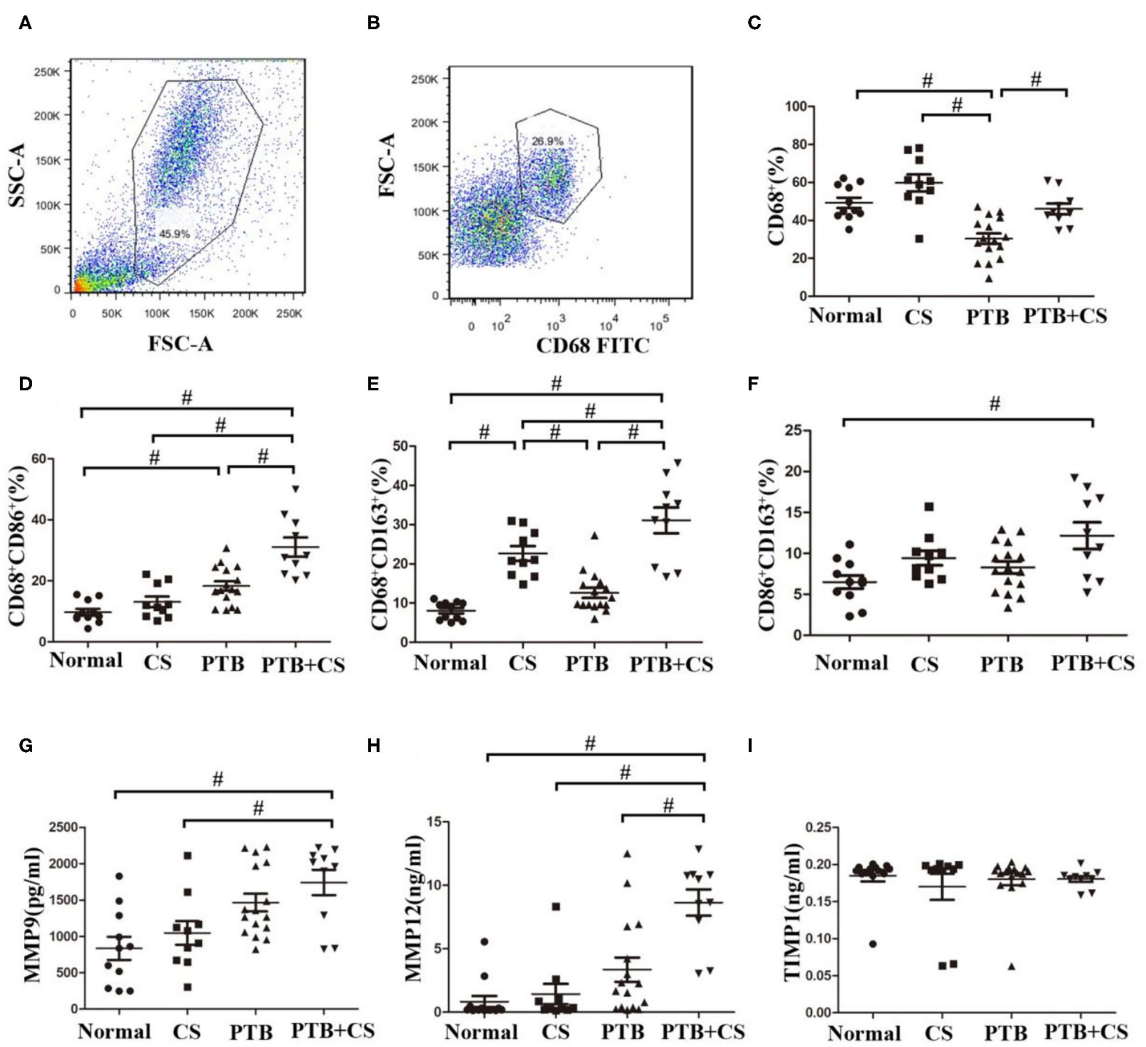
Increased M1 and M2 polarization and MMP production in PTB patients with smoking. **(A)** Macrophage gate in whole cells of BAL fromPTB+CSE, CS, PTB, and Normal groups. **(B)** CD68^+^ cells in macrophage gate. **(C)** The frequencies of macrophages were detected in whole cells of BAL among the four groups. **(D,E)** The frequencies of M1 and M2 macrophages were detected in macrophage gate among the four group. **(F)** The frequencies of CD86^+^CD163^+^ macrophages were detected in CD68^+^ cells among the four group. **(G–I)** The levels of MMP9, MMP12, and TIMP1 were detected in BAL fluid of the four groups. FSC, forward scatter; SSC, side scatter. ^#^*P* < 0.05.

Previous studies had shown that imbalance of macrophage polarization correlated with the development of COPD and PTB (12, 13). We therefore firstly investigated the frequencies of phenotypes of lung macrophages in BAL from the four groups of subjects by surface staining of CD68 (macrophage marker), CD68CD86 (M1 macrophage marker), and CD68CD163 (M2 macrophage marker) (28–30). Our FCM results (Figures 1A–C and Supplemental Figure 1) showed that CD68^+^ macrophage frequencies in BAL cells were highest in the CS group among the four groups. Interestingly, CD68^+^ macrophage frequencies in the PTB+CS group were higher than those in the PTB group, suggesting that CS triggered the recruitment of more macrophages into the lung. Macrophage phenotyping showed that the percentages of both M1 (CD68^+^CD86^+^) and M2 (CD68^+^CD163^+^) macrophages, assessed in macrophage gate (31), were highest in the PTB+CS group among the four groups of subjects (Figures 1D,E). Between-group analysis also showed that the percentage of M2 macrophages was higher in the CS group as compared to the PTB group and the Normal Control, while that of M1 macrophages was higher in the PTB group as compared to the CS group and the Normal Control. Besides, the percentage of double-positive macrophages (CD86^+^CD163^+^), assessed in CD68^+^ cells from macrophage gate, was highest in the PTB+CS group, and significantly higher as compared to the Normal Control (Figure 1F). These results indicated increased M1 and M2 polarization of lung macrophages in PTB patients with smoking, but predominantly M1 phenotype in smokers without PTB and M2 phenotype in PTB patients who did not smoke.

Besides, BAL fluid samples were used to detect the levels of MMPs (MMP9 and MMP12) and their inhibitor TIMP1 by ELISA. The results showed that the levels of both MMP9 and MMP12 were highest in the PTB+CS group among the groups (Figures 1G,H), while TIMP1 showed no difference (Figure 1I), indicating increased MMP9 and MMP12 protein levels in PTB patients who smoked, which may be involved in exaggerated lung tissue damage in these patients.

### BCG Infection Promoted ROS Production, Apoptosis and Expression of M1- and M2 -Related Cytokines in CSE-treated MH-S Cells

**Figure 2:**
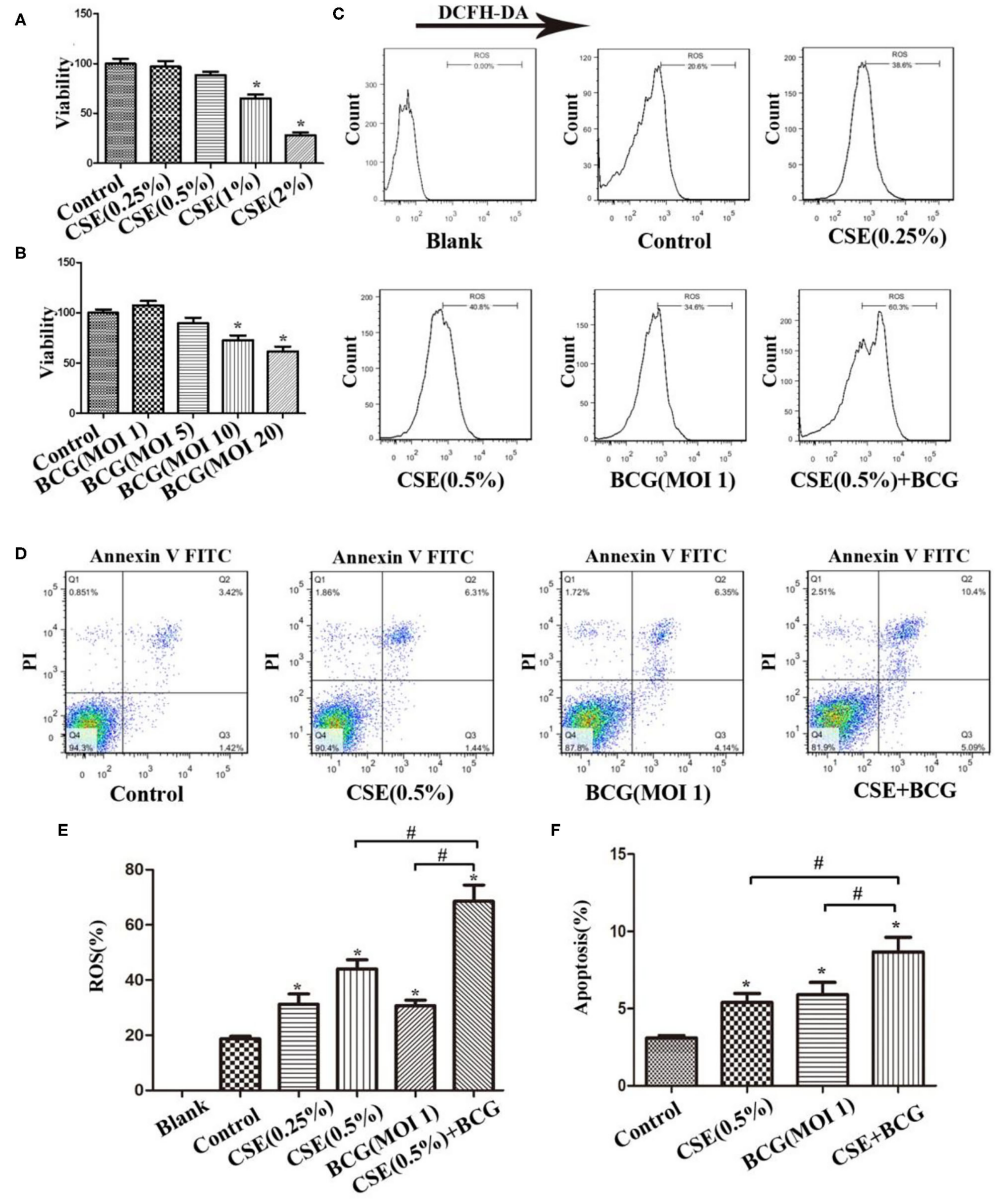
BCG infection promoted ROS production and apoptosis in CSE-treated MH-S cells. **(A)** MH-S cells were treated with CSE for 48 h and cellular viability was assessed. **(B)** MH-S cells were treated with BCG for 48 h and cellular viability was assessed. **(C)** MH-S cells were incubated with various concentrations of CSE for 1 h and then BCG was added for 4 h. ROS level was detected by flow cytometry. **(D)** MH-S cells were incubated with 0.5% CSE for 1 h and then BCG was added for 48 h. Apoptosis of cells was detected by flow cytometry. **(E)** Representative analysis plot of ROS levels. **(F)** Representative analysis plot of cell apoptosis. *P < 0.05 vs. Normal Control group, ^#^*P* < 0.05.

In order to confirm the findings of M1/M2 polarization and MMP production in PTB patients with smoking, we performed the following experiments in an *in vitro* culture system. MH-S cells were stimulated with various concentrations of CSE or BCG for 48 h, and in this system, CSE (<1%) or BCG (MOI ≤ 5) had no effect on cellular viability as determined by MTT assay (Figures 2A,B). FCM results showed that CSE induced concentration-dependent ROS production in MH-S cells, while BCG infection further enhanced CSE-induced ROS production (Figures 2C,E). Besides, annexin V^+^/PI^+^ cells were higher in the CSE+BCG group than those in the CSE or BCG group (Figures 2D,F), suggesting that BCG infection aggravated CSE-induced apoptosis of MH-S cells.

**Figure 3:**
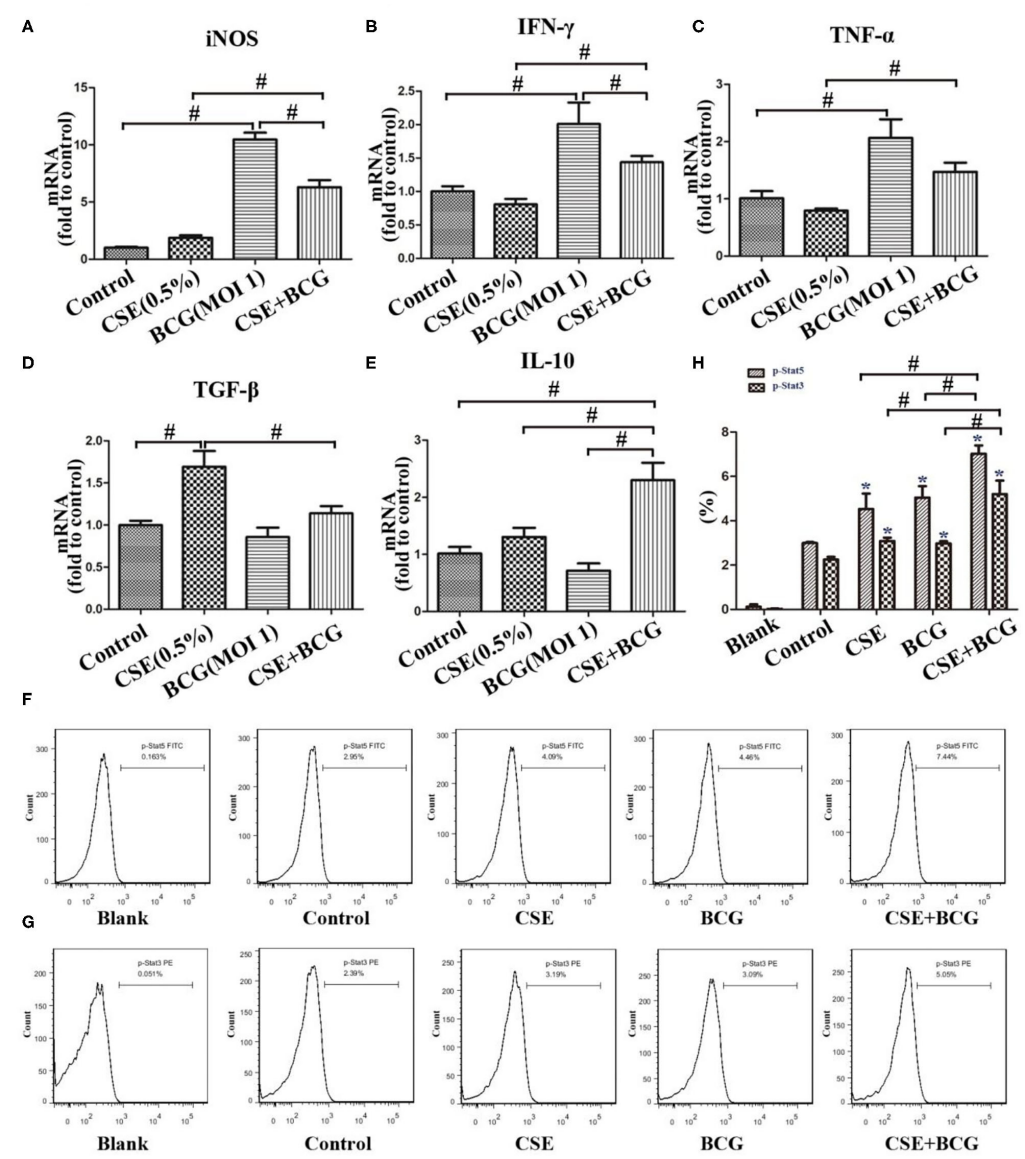
BCG infection promoted expression of M1- and M2 -related cytokines in CSE-treated MH-S cells. **(A–E)** MH-S cells were incubated with 0.5% CSE for 1 h and then BCG was added for 24 h. mRNA expressions of iNOS, IFN-γ, TNF-α, TGF-β, and IL-10 were assessed by qPCR. **(F,G)** MH-S cells were incubated with 0.5% CSE for 1 h and then BCG was added for 30 min. p-Stat5 FITC and p-Stat3 PE were detected by flow cytometry. **(H)** Representative analysis plot of p-Stat5 and p-Stat3. *P < 0.05 vs. Normal Control group. ^#^*P* < 0.05.

Meanwhile, M1-related (iNOS, IFN-γ, and TNF-α) and M2-related cytokines (TGF-β and IL-10) were examined by qPCR after MH-S cells were exposed to CSE (0.5%) and BCG (MOI = 1) for 24 h (Figures 3A–E). BCG infection alone induced increased production of iNOS, IFN-γ, and TNF-α, but had no effect on TGF-β and IL-10, while CSE alone induced up-regulation of TGF-β without effect on other cytokines. Notably, BCG infection in CSE-treated cells showed significantly higher levels of iNOS, IFN-γ, TNF-α, and IL-10, indicating that BCG infection promoted mRNA expression of M1-related and M2-related cytokines in CSE-incubated MH-S cells. Besides, FCM results showed that p-Stat5 and p-Stat3 levels were highest in the CSE+BCG group among the groups (Figures 3F–H), indicating activation of M1-related and M2-related signaling pathways (32).

### BCG Infection Promoted Production of MMP in CSE-Treated MH-S Cells

**Figure 4:**
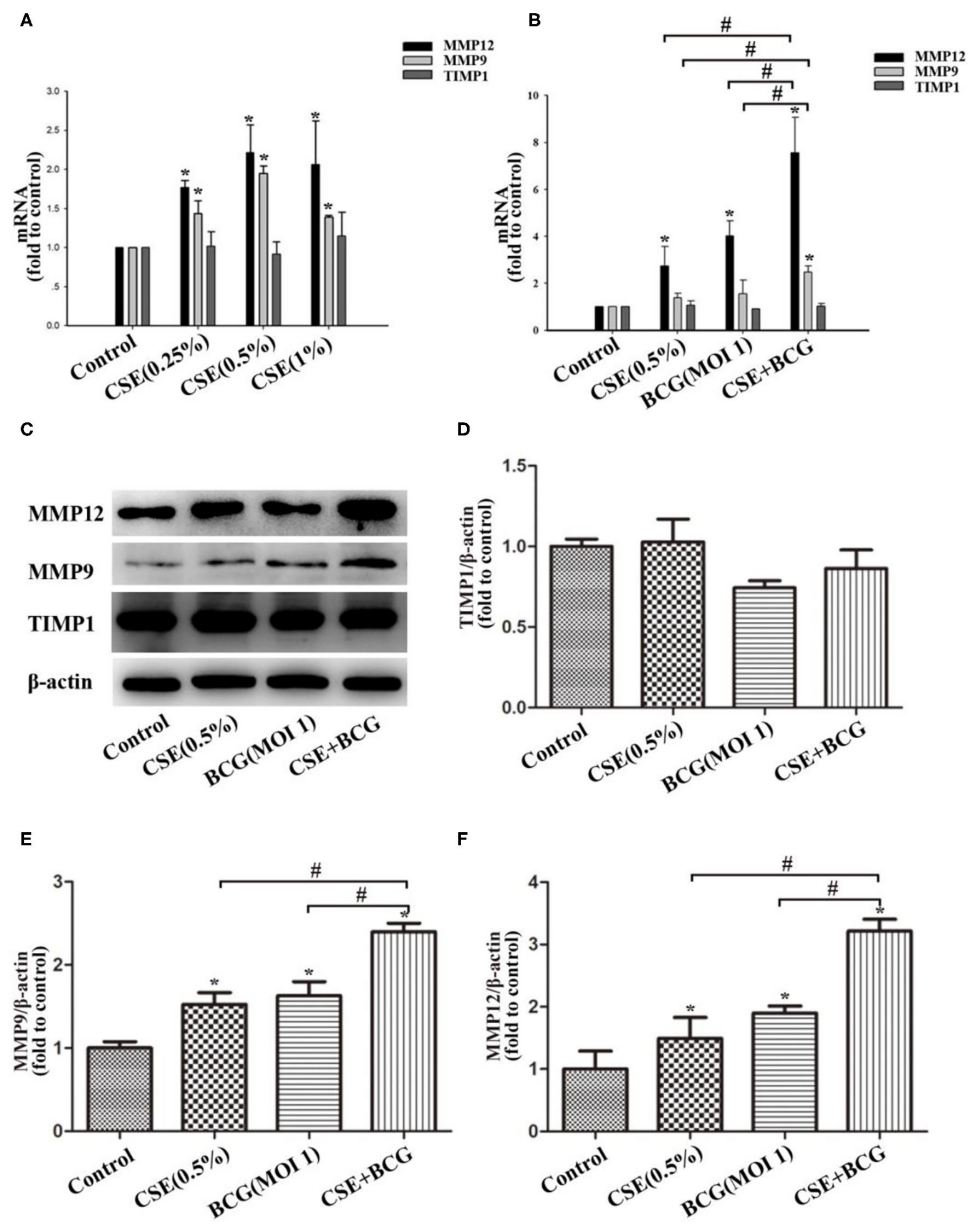
BCG infection promoted the production of MMPs in CSE-treated MH-S cells. **(A)** MH-S cells were treated with CSE for 48 h, and mRNA expressions of MMP9, MMP12 and TIMP1 were assessed by qPCR. **(B)** MH-S cells were incubated with 0.5% CSE for 1 h and then BCG was added for 48 h. and mRNA expressions of MMP9, MMP12 and TIMP1 were assessed by qPCR. **(C–F)** MH-S cells were incubated with 0.5% CSE for 1 h and then BCG was added for 48 h. Protein expressions of MMP9, MMP12, and TIMP1 were assessed by Western blot. *P < 0.05 vs. Normal Control group, ^#^*P* < 0.05.

Then we would like to confirm the production of MMPs in CSE and/or BCG incubated MH-S cells. As expected, CSE induced concentration-dependent mRNA expressions of MMP9 and MMP12, but not TIMP1 (Figure 4A). After pretreated by CSE (0.5%) for 1 h, MH-S cells were infected by BCG (MOI=1) for 48 h. The mRNA and protein levels of MMP9, MMP12, and TIMP1 were assessed by qRCR (Figure 4B) and Western blot (Figures 4C–F). Our results showed that CSE alone or BCG alone did induce high expression of MMP9 and MMP12 mRNA and protein than Control group, while their mRNA and protein levels reached the highest in the CSE+BCG group among the experimental groups. TIMP1 mRNA and protein levels showed no significant difference among groups.

### Exaggerated M1 and M2 Polarization and MMP Production in CSE-Exposed Mice With BCG-Infection

**Figure 5:**
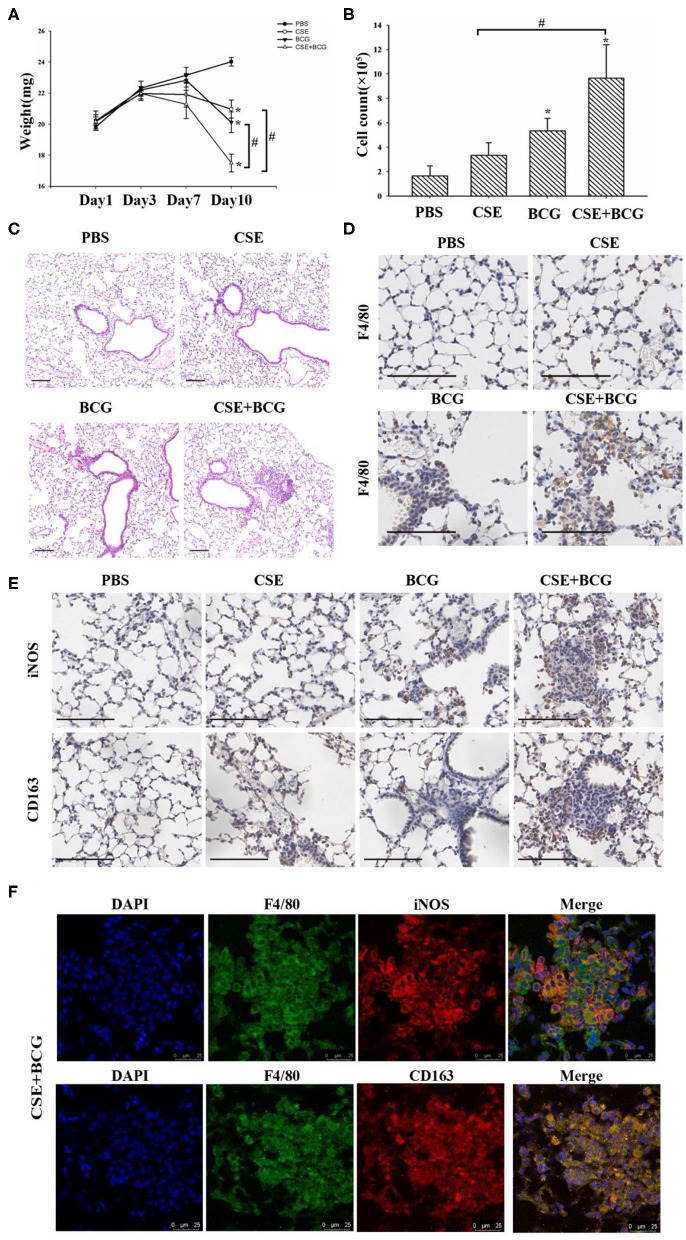
Exaggerated M1 and M2 polarity in CSE-exposed mice with BCG infection. **(A)** Representative changes of weight. *N* = 8 per group. **(B)** Total inflammatory cell counts in BAL. *N* = 6 per group. **(C)** Representative H&E-stained lung sections. Bar 100 μm. *N* = 8 per group. **(D)** Representative immunohistochemistry of F4/80 in lung tissues of mice. Bar 100 μm. *N* = 8 per group. **(E)** Representative immunohistochemistry of iNOS and CD163 in lung tissues of mice. Bar 100 μm. *N* = 8 per group. **(F)** Representative immunofluorescence of F4/80, iNOS and CD163 in lung tissues of mice. Bar 25 μm. *N* = 8 per group.*P < 0.05 vs. PBS group, ^#^*P* < 0.05.

**Figure 6:**
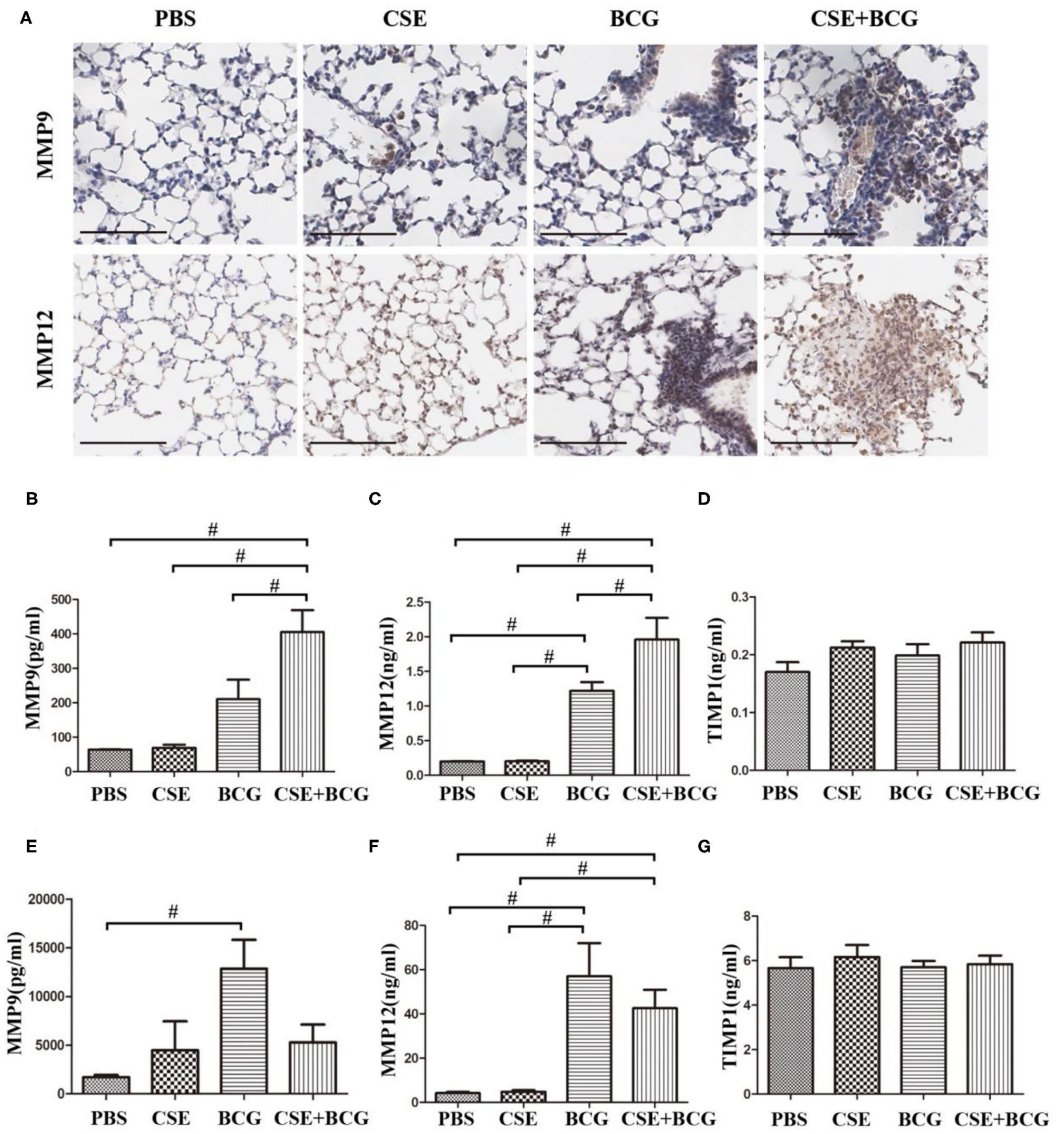
Enhanced MMP production in CSE-exposed mice with BCG infection. **(A)** Representative immunohistochemistry of MMP9 and MMP12 in lung tissues of mice. Bar 100 μm. *N* = 8 per group. **(B–D)** The levels of MMP9, MMP12, and TIMP1 were detected in BAL fluid of mice. *N* = 6 per group. **(E–G)** The level of MMP9, MMP12, and TIMP1 were detected in serum of mice. *N* = 8 per group. ^#^*P* < 0.05.

In order to confirm the findings of M1/M2 polarization and MMP production *in vivo*, we exposed mice to short-term CSE and then infected with or without BCG. Our results showed that the loss of body weight and the increase of inflammatory cells in BAL were most prominent in CSE-exposed mice with BCG infection (Figures 5A,B), which also showed the most marked inflammatory lesions in the lung (Figure 5C). Immunohistochemistry results revealed large number of F4/80 positive cells in lung tissues of mice with CSE exposure and BCG infection (Figure 5D), indicating recruitment and infiltration of macrophages. Higher expressions of iNOS and CD163 were observed in lung lesions of CSE-exposed mice with BCG-infection (Figure 5E), where co-localization of iNOS or CD163 with F4/80 were demonstrated (Figure 5F), suggesting presence of M1 and M2 polarized macrophages in lung lesions. Besides, MMP9 and MMP12 were highly expressed in lung lesions in CSE-exposed mice with BCG-infection (Figure 6A). Consistently, levels of MMP9 and MMP12, but not TIMP1, in BAL fluid were highest in the CSE+BCG mice as compared to mice exposed to PBS, CSE alone or BCG alone (Figures 6B–D). However, the serum levels of these proteinases were not different among groups (Figures 6E–G).

## Discussion

In the present study, for the first time to our knowledge, we found increases in both M1 and M2 macrophages and higher levels of MMP9 and MMP12 in BAL fluid from PTB patients who smoked. We also confirmed *in vitro* that BCG infection in CSE-incubated MH-S cells further enhanced the expression of M1-related (iNOS, IFN-γ and TNF-α) and M2-related cytokines (TGF-β and IL-10), ROS production, cellular apoptosis and MMP (MMP9 and MMP12) production. These novel findings were largely recapitulated in a mouse model of short-term CSE exposure after infection with BCG, which also induced exacerbated inflammation in the lung. We believe these results would shed light on mechanims underlying persistent inflammation and tissue destruction seen in COPD associated with PTB.

Besides cigarette smoking, PTB is considered as an important risk factor for COPD, especially in TB high-burden regions. A cross-sectional study in Guangzhou found that the prevalence of prior TB in 8,066 participants, as defined by chest radiographs, was 24.2%, and prior TB was an independent risk factor for airflow obstruction (5). Aggarwal and colleagues found that 32.4% of COPD patients had a history of TB, evaluated through self-reporting and/or checking previous records (33). A study in Latin America also showed that history of tuberculosis was associated with airflow obstruction in middle-aged and older adults (3). Our previous work found that the percentage of previous PTB in COPD patient was 45% by high-resolution computed tomography (HRCT), and was associated with more severe lung damage and emphysema (6). A self-reported history of tuberculosis was associated with both airflow obstruction and spirometric restriction in the multicentre, cross-sectional, general population-based Burden of Obstructive Lung Disease study (4). Other studies also indicated that history of previous tuberculosis was associated with more severe form of COPD, evaluated by CAT scores, lung function, mortality, and exacerbation prevalence (6, 34–36). However, the cellular and molecular mechanisms underlying the interplay between Mtb and CS exposure in the pathogenesis or progression of COPD are largely unknown.

Macrophages play a critical role in regulation of innate and acquired immunity, which can potentiate inflammation and trigger an immune response. Macrophages are increased with activated function in lung tissues and BAL of COPD, which may responsible for pathogenesis of the disease (14, 37). Under certain stimuli such as LPS, macrophages are driven toward two major polarized phenotypes: M1 and M2, which exert pro-inflammation and anti-inflammation functions, respectively (7, 10, 38, 39). Studies in COPD patients and mouse models have implicated macrophage polarization in pathogenesis of the disease (7, 12, 14, 30). CS can induce an M2 polarization program, with deactivation of M1 polarization program, in alveolar macrophage (40). Bazzan et al. reported that M1 and M2 polarization increased significantly with smoking and COPD severity and so did the co-expression of M1 and M2 molecules in the same alveolar macrophage (12). On the contrary, Mtb infection can drive macrophages toward M1 phenotypes in early phase, and shift their program into M2 phenotypes in intermediate and late phases (13, 41). However, lung macrophage phenotypes had not been specifically investigated in smoking patients with PTB. We believe that a better understanding of macrophage polarization under CS and Mtb co-exposure should yield novel information on Mtb-related lung damage in COPD pathogenesis. In line with previous studies, we confirmed here that M2 polarized macrophages were programmed in smokers without PTB (CS group), while M1 polarized macrophages were prevalent in PTB patient who did not smoke (PTB group). Notably, both M1 macrophages and M2 macrophages were highest in the lung of PTB patients who smoked (PTB+CS group). Double positive macrophages (CD86^+^CD163^+^) were highest in PTB patients who smoked, suggesting co-expression of M1 and M2 markers in the lung macrophages. Therefore, it seems likely that Mtb infection promoted CS-induced M2 macrophages to shift into both M1 and M2 programs, contributing to bacterial killing on one hand and exaggerated airway inflammation and lung destruction on the other.

A previous study demonstrated that CSE reduced the expression of IFN-γ, TNF-α, and IL-10 responses in BCG-driven macrophages, impairing bacterial containment (42). Our results here showed that, though the expressions of iNOS, IFN-γ, TNF-α, and TGF-β in macrophages were decreased in the CSE+BCG group as compared to the BCG group, but were still significantly higher in the CSE+BCG group as compared to the CSE group, suggesting promotion by BCG infection on M1-related and M2-related cytokines in CSE-treated macrophages. Meanwhile, ROS, another related marker of M1 phenotype (39), was induced by CSE alone or BCG alone, but was the highest in CSE+BCG group, suggesting that BCG infection promoted CSE-induced ROS production. Our mouse model also revealed that M1 and M2 macrophages were found in lung lesions of acutely CSE-exposed mice with BCG infection. Moreover, p-Stat5 and p-Stat3 levels were highest in CSE exposed MH-S cells with BCG infection, suggesting activation of M1-related and M2-related signaling pathways. Therefore, these results indicated in *vitro* and *in vivo* that BCG infection promoted CSE-induced macrophage polarization toward both M1 and M2 programs, characterized by their related effectors and signals. However, we still wondered why not all markers of polarization were up-regulated with the increase of both M1 and M2 macrophages in the CSE+BCG group. Recent studies found that M1 macrophages could be further classified to subtypes of M1a and M1b (43), while M2 macrophages could be subdivided into at least three different subsets, namely, M2a, M2b, and M2c (13, 32). A better understanding of these newer subsets of macrophages in our experimental model would answer this question. In the current study, we also found that apoptosis of macrophages in the CSE+BCG group was increased as compared to the CSE group and the BCG group, indicating further impairment of the capability of macrophages in controlling Mtb infection.

MMPs, such as MMP9 and MMP12, are the major effector molecules of lung damage in COPD and PTB, and they are mostly produced by pulmonary macrophages (21, 23). However, whether co-exposure to CS and Mtb has a synergistic effect on the MMP production is unknown. Our results in human BAL fluid revealed that the levels of MMP9 and MMP12, but not TIMP1, were highest in the PTB+CS group than those in the other groups. Subsequent experiments with MH-S cells and the mouse model confirmed that BCG infection enhanced CSE-induced production of MMP9 and MMP12. However, the relationship between macrophage polarization and MMP production is still complex and controversial. Several studies demonstrated that it was M1 macrophages that produced MMP9 and MMP12, while others found that MMP9 and MMP12 belonged to products of M2 macrophages (44–47). Another study also showed that MMP12 appeared to be more readily up-regulated in M2 macrophages than M1 macrophages (48). Therefore, the exact relation between enhanced MMP production and M1/M2 polarization in our study still needs further investigation.

Our study had several limitations. The PTB patients we enrolled were smokers, but not patients with COPD, because enrollment of COPD patients with active tuberculosis was difficult. However, our patients had a history of considerable amount of smoking, which should be adequate to illustrate the combined effect of CS exposure and Mtb infection. Because patients with PTB and subjects in the control groups were consecutively enrolled when they had clinical indications for bronchoscopy, the age among groups was different, which may be a confounding variable, although the younger age of PTB patients was in line with epidemiological characteristics. Another limitation was that we used acute exposure to CS in the animal model, which was though appropriate for the purpose of the study, but not as relevant as a COPD model by long-term CS exposure.

In summary, by carefully designed studies in human patients, animal models and *in vitro* experiments, we demonstrated that Mtb infection promoted CS-exposed macrophages to polarize toward both M1 and M2 phenotypes, along with enhanced production of MMP9 and MMP12. These findings provide insights into the mechanistic interactions between CS exposure and tuberculosis in the pathogenesis of COPD.

## Data Availability Statement

All datasets presented in this study are included in the article/Supplementary Material.

## Ethics Statement

The studies involving human participants were reviewed and approved by the Ethics Committee of Peking University Third Hospital and Beijing Geriatric Hospital. The patients/participants provided their written informed consent to participate in this study. The animal study was reviewed and approved by the Ethics Committee of Peking University Third Hospital.

## Author Contributions

YL, BW, and YS designed the study and experiments. YL, WC, QL, FL, XG, CC, AL, WX, BL, and TW recruited the patients, collected samples and analyzed the clinical data. LZ, XF, JX, and YR analyzed data. YL and YS wrote the manuscript. All authors contributed to the article and approved the submitted version.

## Conflict of Interest

The authors declare that the research was conducted in the absence of any commercial or financial relationships that could be construed as a potential conflict of interest.
