# Supplementary figures and images for: Infection of Mycobacterium tuberculosis Promotes Both M1/M2 Polarization and MMP Production in Cigarette Smoke-Exposed Macrophages
Source: Front Immunol. 2020 Aug 20;11:1902. doi: 10.3389/fimmu.2020.01902 (PMC7468417; doi:10.3389/fimmu.2020.01902)

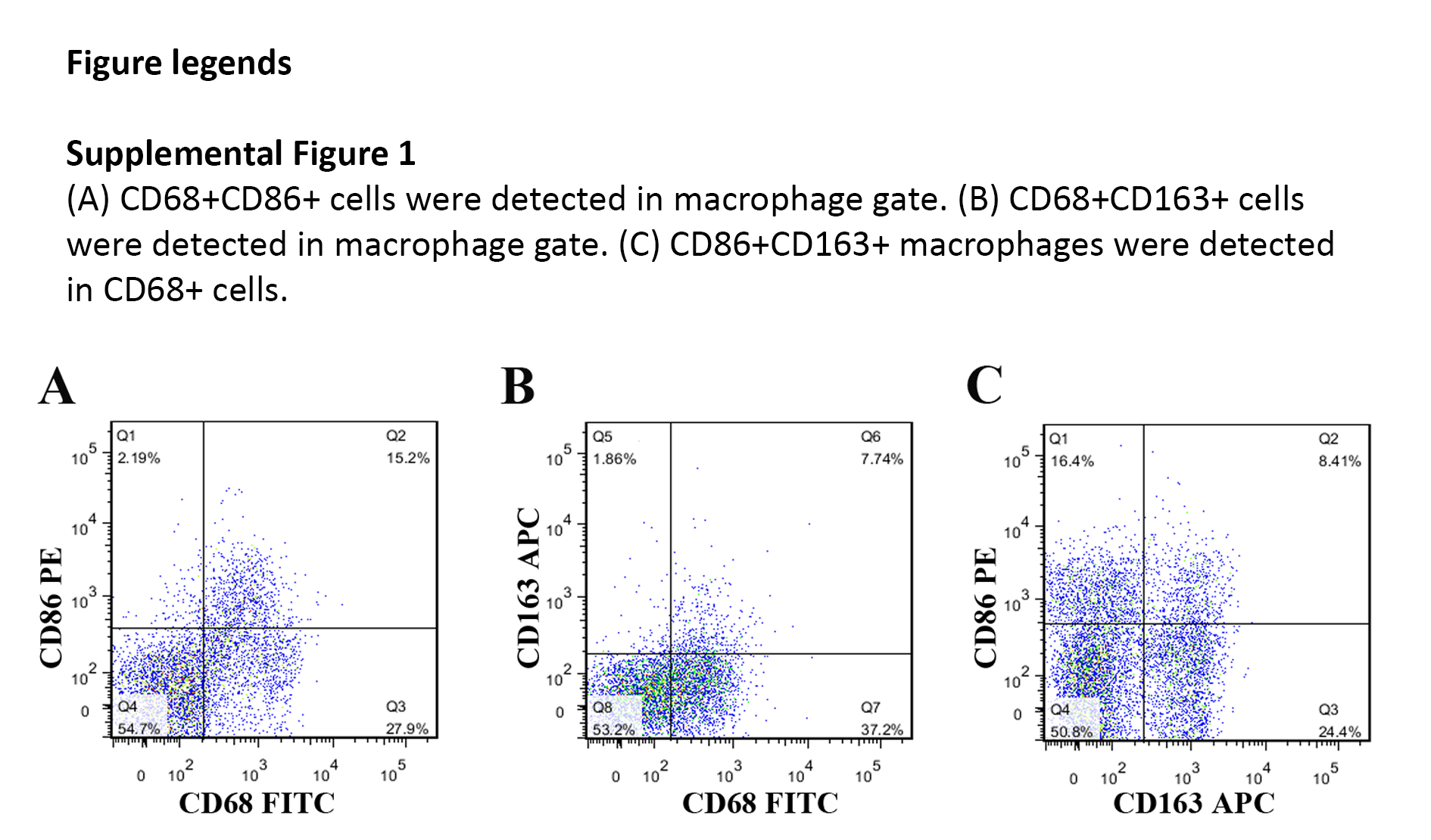

Supplement: Supplementary file 1 [file Image_1.tif]
